# Associations of alarm fatigue with occupational burnout and sleep quality in ICU nurses: a cross-sectional study

**DOI:** 10.1186/s12912-026-05165-y

**Published:** 2026-08-03

**Authors:** Somayeh Zeidabadinejad, Reza Sadeghi, Parniya Abolghaseminejad, Iman Nosratabadi, Leyla Ahmadi Lari, Fatemeh Yousefi Baseri

**Affiliations:** 1 Student Research Committee, Department of Anesthesiology, Sirjan School of Medical Sciences, Sirjan, Iran; 2 Department of Public Health, Sirjan School of Medical Sciences, Sirjan, Iran; 3 Department of Health Education and Promotion, School of Public Health, Tehran University of Medical Sciences, Tehran, Iran; 4 Department of Nursing, Sirjan School of Medical Sciences, Sirjan, Iran; 5 Department of Anesthesiology, School of Nursing, Larestan University of Medical Sciences, Larestan, Iran; 6 Student Research Committee, Sirjan School of Medical Sciences, Sirjan, Iran

**Keywords:** Alarm fatigue, Occupational burnout, Sleep quality, ICU nurses

## Abstract

**Background:**

Alarm fatigue is a critical concern in intensive care units (ICUs), where nurses are continuously exposed to high volumes of clinical alarms. This phenomenon has been associated with psychological strain, impaired sleep quality, and occupational burnout. Despite growing evidence, limited studies have simultaneously explored the associations among these variables in ICU nurses. This study aimed to investigate the relationships among alarm fatigue, occupational burnout, and sleep quality in ICU nurses working in hospitals in Kerman and Sirjan, Iran.

**Methods:**

A descriptive–analytical cross-sectional study was conducted among 219 ICU nurses selected through census sampling. Data were collected using a demographic questionnaire, the Alarm Fatigue Scale (AFSS), the Maslach Burnout Inventory (MBI), and the Pittsburgh Sleep Quality Index (PSQI). Statistical analyses were performed using SPSS version 22, employing independent t-test, ANOVA, Mann–Whitney U, Kruskal–Wallis, Spearman’s correlation coefficient, and multivariable linear regression analyses. A significance level of *p* < 0.05 was considered.

**Results:**

More than half of the nurses reported no alarm fatigue (57.5%), while 83.6% experienced poor sleep quality. Emotional exhaustion was low to moderate in most participants, depersonalization was moderate in 56.2%, and reduced personal accomplishment was reported by 96.3%. Alarm fatigue showed weak but statistically significant positive correlations with poor sleep quality (*r* = 0.230, *p* = 0.001) and occupational burnout (*r* = 0.166, *p* = 0.014). Sleep quality was also weakly but significantly correlated with occupational burnout (*r* = 0.175, *p* = 0.010). Alarm fatigue and burnout were significantly associated with gender, employment status, shift type, and general health status.

**Conclusions:**

Alarm fatigue, poor sleep quality, and occupational burnout are related occupational health concerns among ICU nurses. Higher alarm fatigue was weakly but significantly associated with poorer sleep quality and greater burnout. These findings suggest that alarm management, shift scheduling, and workforce-related factors may be relevant areas for organizational attention; however, future longitudinal and interventional studies are needed to determine whether such strategies improve sleep quality, reduce burnout, or enhance patient safety.

**Clinical trial number:**

Not applicable.

## Background

Alarm fatigue has emerged as a growing global concern in public health and patient safety [1]. The American Association of Critical Care Nurses defines alarm fatigue as a form of sensory overload that occurs when clinicians are exposed to excessive alarm signals, resulting in desensitization to alarm sounds and an increased likelihood of missed alarms [2]. This phenomenon is particularly prevalent in intensive care units (ICUs), where critically ill patients depend on multiple monitoring devices to detect physiological changes [3]. Although these devices are essential for timely clinical intervention, studies indicate that 85%–99% of alarms are false or clinically insignificant [4]. As reported, “During a single shift, healthcare personnel may be exposed to as many as 1000 device alarms” [5]. Such excessive non-actionable alarms consume nurses’ time and energy, disrupt patient care, and diminish trust in alarm systems [6].

International and professional guidance increasingly frames clinical alarm management as both a patient-safety and human-factors issue rather than a purely technical problem. The Joint Commission’s National Patient Safety Goal NPSG.06.01.01 emphasizes reducing patient harm associated with clinical alarm systems by establishing alarm safety as an organizational priority, identifying the most important alarm signals to manage, and developing policies regarding alarm settings, disabling alarms, changing alarm parameters, monitoring alarm signals, and checking alarm detectability [7]. Similarly, the American Association of Critical-Care Nurses recommends evidence-based alarm management practices, including proper ECG electrode and pulse oximetry probe placement, checking alarm settings at the beginning of each shift and after changes in patient condition or caregivers, customizing alarm parameters for individual patients according to unit policy, forming interprofessional alarm-management teams, developing unit-specific default parameters, and providing ongoing staff education [8]. The AAMI Clinical Alarm Management Compendium also highlights that alarm-management strategies should be locally adapted, supported by benchmarking of alarm parameters, staff education, and continuous quality improvement rather than implemented as one-size-fits-all solutions [9]. Recent human-factors research further emphasizes that alarm management in ICUs is embedded within complex clinical workflows and that sustainable solutions should be designed with users, real-world workflows, and alarm data at the core [10, 11].

The reported level of alarm fatigue has varied across different studies. In some studies, nurses have been found to experience low levels of alarm fatigue [12], whereas in others, nurses report high levels of alarm fatigue [13, 14]. Factors associated with alarm fatigue are multifactorial, encompassing patient characteristics, device-related factors, work environment conditions, and organizational elements [15]. Personal factors such as anxiety, stress, and personality traits have also been associated with alarm fatigue [16]. Recent evidence demonstrates a significant association between alarm fatigue and occupational burnout among critical care nurses [1, 13, 17]. Burnout is defined as a state of emotional, physical, and psychological exhaustion resulting from prolonged unmanaged work-related stress [18]. Due to the demanding nature of intensive care nursing, ICU nurses are at particularly high risk for burnout [19], which is associated with poorer well-being, reduced job performance, adverse organizational outcomes, and compromised patient safety [20, 21]. In various Iranian and international studies, the level of job burnout has been reported to range from moderate [12, 22] to high [23]. Furthermore, the occupational characteristics of nursing, especially rotating shifts and heavy workloads, substantially increase the likelihood of insufficient and poor-quality sleep [24, 25]. Sleep disturbance, as a stress-inducing condition, triggers the release of catecholamines and leads to increased respiration, blood pressure, and myocardial oxygen demand, ultimately resulting in an increased cardiac workload [26]. Adequate sleep is essential for hormonal regulation, energy restoration, memory consolidation, and cognitive functioning [27, 28]. Poor sleep quality is characterized by frequent nocturnal awakenings, reduced sleep efficiency, and impaired daytime cognitive performance [29]. As noted, “Insufficient sleep negatively impacts nurses’ concentration and judgment, potentially resulting in mistakes and injuries” [24]. ICU nurses, who manage complex procedures and critically ill patients, are particularly vulnerable to sleep disturbances [30–32]. Insomnia is further characterized by prolonged sleep onset latency, repeated awakenings, and sustained wakefulness during the night [33]. A review of existing evidence indicates significant pairwise associations between alarm fatigue and occupational burnout [12, 13], alarm fatigue and sleep disturbances [26], and occupational burnout and sleep disorders [34, 35]. However, most previous studies have examined these associations separately, which limits understanding of how these occupational and health-related factors co-occur within the same group of ICU nurses. Simultaneously assessing alarm fatigue, sleep quality, and occupational burnout may provide a more comprehensive description of nurses’ work-related strain by clarifying whether these problems are isolated or occur together as related dimensions of occupational well-being. Therefore, the present study sought to examine the associations among these three variables in a single sample of ICU nurses, without assuming causal directionality.

The conceptual rationale of this study was informed by the Job Demands–Resources (JD-R) model [36, 37]. According to this model, demanding aspects of the work environment may be associated with strain-related outcomes, particularly when they require sustained physical, cognitive, or emotional effort [36, 37]. In ICU settings, frequent exposure to clinical alarms can be considered a specific job demand because nurses are continuously exposed to high volumes of alarms and must maintain attention to alarm signals in order to ensure patient safety [1, 5, 15]. Within this framework, alarm fatigue may reflect nurses’ perceived burden of alarm exposure, whereas poor sleep quality and occupational burnout represent health- and strain-related outcomes [20, 34–37]. However, given the cross-sectional design of the present study, the JD-R model was used only as a conceptual framework to guide variable selection and interpretation, not to test causal or mediating pathways [38].

Given that human resources, particularly nurses, represent the most valuable assets of healthcare organizations, ensuring their physical and psychological well-being is essential for enhancing organizational productivity and efficiency [39]. Understanding the consequences of alarm fatigue in clinical settings is crucial, as it may improve patient comfort and prevent potential complications. Likewise, the relationship between occupational burnout and sleep quality poses an important challenge that must be addressed to support nurses’ overall health. Investigating the association between alarm fatigue, occupational burnout, and sleep quality among ICU nurses is therefore of great importance. Frequent exposure to alarms may be associated with higher perceived stress and fatigue, poorer sleep quality, lower psychological well-being, and greater burnout; however, the direction and causal nature of these associations require further longitudinal investigation. Nurses experiencing high stress and inadequate sleep are less capable of providing effective patient care. Addressing these issues may help reduce workplace stressors, improve working conditions, and ultimately enhance patient safety and care quality.

## Methods

### Study design and setting

This descriptive–analytical cross-sectional study was conducted among intensive care unit (ICU) nurses working in four hospitals in Kerman and Sirjan, Iran (Shafa, Afzalipour, Bahonar, and Samen Al-Hojaj). Guided by the JD-R model, this study aimed to examine the associations among alarm fatigue, sleep quality, and occupational burnout among ICU nurses.

### Participants and sampling

The study population included all ICU nurses employed in the selected hospitals. A census sampling method was used due to the limited number of eligible nurses. Inclusion criteria were: holding a nursing degree, at least one year of full-time ICU work experience, and willingness to participate. Nurses who did not complete the questionnaires were excluded.

Of the 300 eligible ICU nurses approached through census sampling, 219 completed the questionnaires and were included in the final analysis, yielding a response rate of 73.0%. The remaining 81 nurses either declined participation or returned incomplete questionnaires and were therefore excluded. Because demographic and occupational information was not available for nonrespondents, comparisons between respondents and nonrespondents could not be performed. Therefore, the possibility of nonresponse bias cannot be excluded.

### Data collection instruments

#### Demographic questionnaire

This form collected information on age, gender, marital status, education level, employment status, work shift, ICU work experience, and monthly working hours.

#### Alarm Fatigue Scale (AFSS)

The 10-item Alarm Fatigue Scale developed and validated by Dehghan et al. [8] was used to assess alarm fatigue. The scale includes two subscales (psychosomatic symptoms and care-related symptoms) scored on a 5-point Likert scale (0 = never to 4 = always). Total scores range from 0 to 40, categorized as:

- 0–10: no alarm fatigue
- 11–20: mild
- 21–30: moderate
- 31–40: moderate to severe
- Cronbach’s alpha for the original scale ranged from 0.85 to 0.875 [12].

In the present study, the internal consistency of the AFSS was good, with a Cronbach’s alpha of 0.870 for the total scale.

#### Maslach Burnout Inventory (MBI)

Occupational burnout was measured using the 22-item Maslach Burnout Inventory [40], consisting of three subscales:

- Emotional exhaustion
- Depersonalization
- Personal accomplishment
- Items are scored on a 7-point Likert scale (0–6). Higher scores on emotional exhaustion and depersonalization indicate greater burnout, whereas lower scores on personal accomplishment indicate reduced professional efficacy and a higher level of burnout in this dimension. Reliability coefficients reported by Maslach and Jackson were 0.90, 0.70, and 0.71 for the three subscales, respectively [35]. Validity and reliability have also been confirmed in Iranian studies [41].

In the current sample, Cronbach’s alpha coefficients for the MBI subscales were 0.855 for emotional exhaustion, 0.555 for depersonalization, and 0.711 for personal accomplishment. These results indicated good internal consistency for emotional exhaustion, acceptable internal consistency for personal accomplishment, and relatively low internal consistency for depersonalization.

#### Pittsburgh sleep quality index (PSQI)

Sleep quality was assessed using the 19-item PSQI developed by Buysse et al. [42]. The instrument includes seven components, each scored from 0 to 3, yielding a total score of 0–21. Scores > 5 indicate poor sleep quality. The original scale demonstrated a Cronbach’s alpha of 0.83 [42], and the Persian version showed an alpha of 0.79 [43].

In the present study, the PSQI demonstrated good internal consistency, with a Cronbach’s alpha of 0.852.

### Study procedure

Data were collected through a descriptive–analytical cross-sectional study conducted among ICU nurses working in hospitals in Kerman and Sirjan, Iran. After obtaining ethical approval and institutional permissions, the researcher visited the ICU wards across different work shifts (morning, evening, and night). Eligible nurses were informed about the study objectives and procedures, inclusion/exclusion criteria, confidentiality of information, and the voluntary nature of participation. The use of verbal informed consent was approved by the Ethics Committee of Sirjan School of Medical Sciences.

Data collection was carried out using printed and electronic questionnaires based on participants’ preference. Completing the instruments took approximately 10–15 min. All returned questionnaires were checked for completeness prior to data entry.

This study was conducted in accordance with the ethical standards of institutional and national research committees and the principles of the Declaration of Helsinki. Ethical approval was obtained from the Ethics Committee of Sirjan School of Medical Sciences (approval code: IR.SIRUMS.REC.1404.008). Permission to collect data was granted by the nursing management departments and hospital administrators of all participating institutions. No identifying personal information was collected; all data were handled anonymously and used solely for research purposes.

### Statistical analysis

Data were analyzed using SPSS version 22. Descriptive statistics were reported as frequency and percentage for categorical variables and as mean ± standard deviation for quantitative variables. The internal consistency of the study instruments was assessed using Cronbach’s alpha coefficient in the current sample.

The normality of quantitative variables was assessed using the Shapiro–Wilk test and visual inspection of histograms and Q–Q plots. Based on the normality assessment, the occupational burnout total score was approximately normally distributed and was analyzed using parametric tests when appropriate, whereas alarm fatigue and sleep quality did not meet the assumption of normality and were analyzed using nonparametric tests. Accordingly, independent samples t-test and one-way ANOVA were used for normally distributed variables, while Mann–Whitney U and Kruskal–Wallis tests were applied for non-normally distributed variables, as appropriate.

Spearman’s correlation coefficient was used to examine the associations among alarm fatigue, sleep quality, and occupational burnout because at least one of the main study variables did not meet the assumption of normality. To examine whether these associations remained significant after adjustment for potential confounders, multivariable linear regression analyses were performed using the enter method to ensure that the main study variables and theoretically relevant covariates were retained in the adjusted models. Three separate models were fitted with alarm fatigue, sleep quality, and occupational burnout as dependent variables. In each model, the other two main study variables were entered as independent variables, together with demographic and occupational covariates, including age, gender, employment status, work shift, monthly number of shifts, and self-rated general health status. Categorical variables, including employment status and work shift, were entered using dummy coding, with permanent employment and rotating shift as reference categories. Regression coefficients (B), standard errors (SE), standardized coefficients (β), 95% confidence intervals, and p-values were reported. Multicollinearity was assessed using variance inflation factor (VIF), and model assumptions were evaluated through residual analysis. A significance level of *p* < 0.05 was considered for all statistical analyses.

## Results

### Demographic characteristics

A total of 219 ICU nurses participated in the study. As shown in Table 1, most participants were female (67.6%), aged 30–39 years (58.0%), married (63.9%), and held a bachelor’s degree (81.3%). The majority were permanently employed (54.3%) and worked rotating shifts (88.6%).

**Table 1:** Frequency and percentage distribution of demographic characteristics

Variable	Category	Frequency	Percent
Gender	Female	148	67.6
	Male	71	32.4
Age group	< 30	59	26.9
	30–39	127	58.0
	≥ 40	33	15.1
Marital status	Single	79	36.1
	Married	140	63.9
Education	Bachelor’s	178	81.3
	Master’s	41	18.7
Employment status	Permanent	119	54.3
	Contractual	43	19.6
	Temporary	39	17.8
	Trainee	18	8.2
Work shift	Rotating	194	88.6
	Morning	12	5.5
	Evening	13	5.9
General health status	Poor	14	6.4
	Average	104	47.5
	Good	87	39.7
	Very Good	14	6.4

### Descriptive statistics of quantitative variables

As shown in Table 2, the mean age of nurses was 33.4 ± 5.3 years, mean ICU work experience was 3.2 ± 1.1 years, mean monthly shifts were 20.9 ± 8.4, and mean monthly night shifts were 7.1 ± 4.9.

**Table 2:** Descriptive indicators of quantitative variables

Variable	*N*	Mean	SD	Min	Max
Age (years)	219	33.4	5.3	22	45
ICU experience (years)	219	3.2	1.1	1	5
Monthly shifts	218	20.9	8.4	0	64
Monthly night shifts	175	7.1	4.9	0	29

### Distribution of questionnaire scores

Table 3 shows that 57.5% of nurses reported no alarm fatigue, 37.9% mild fatigue, and 4.6% moderate fatigue. Sleep quality was poor in 83.6% of participants. Regarding burnout, most nurses reported reduced personal accomplishment (96.3%), while depersonalization was moderate in 56.2% of participants. Given the very high proportion of reduced personal accomplishment, this finding should be interpreted with caution and considered in relation to contextual and measurement-related factors.

**Table 3:** Distribution of questionnaire scores

Questionnaire	Category	Frequency	Percent
Alarm fatigue	None	126	57.5
	Mild	83	37.9
	Moderate	10	4.6
Sleep quality (PSQI)	Good	36	16.4
	Poor	183	83.6
Emotional exhaustion	Low	112	51.1
	Moderate	80	36.5
	High	27	12.3
Depersonalization	Low	31	14.2
	Moderate	123	56.2
	High	65	29.7
Personal accomplishment	Low	211	96.3
	Moderate	8	3.7
	High	0	0

Although more than half of the nurses reported no alarm fatigue, poor sleep quality was highly prevalent across the sample. This pattern suggests that poor sleep quality may not be attributable only to alarm fatigue and may also reflect other occupational and contextual factors, such as rotating shifts, night work, workload, and the stressful nature of ICU practice.

### Comparison of alarm fatigue scores

As presented in Table 4, alarm fatigue differed significantly across:

**Table 4:** Comparison of alarm fatigue scores across demographic variables

Variable	Category	Mean	SD	Mean Rank	Sig.
Gender	Female	10.4	6.1	120.1	0.001¹
	Male	7.5	6.0	89.0	
Age group	< 30 years	11.5	6.3	130.2	0.001²
	30–39 years	9.5	6.2	108.7	
	≥ 40 years	6.4	5.2	79.0	
Marital status	Single (widowed/divorced included)	9.2	7.1	105.1	0.388¹
	Married	9.6	5.7	112.8	
Education level	Bachelor’s	9.8	6.5	112.5	0.233¹
	Master’s	8.3	4.8	99.4	
Employment status	Permanent	8.8	6.3	102.6	0.003²
	Contractual	8.4	6.5	98.2	
	Temporary	10.9	5.8	126.2	
	Trainee	13.3	4.2	152.1	
Work shift	Rotating	9.9	6.2	114.9	0.001²
	Morning	8.3	6.5	95.5	
	Evening	3.8	3.9	50.2	
General health status	Poor	12.8	7.2	141.4	0.013²
	Moderate	9.0	6.6	103.6	
	Good	10.1	5.4	118.5	
	Very good	6.1	6.0	73.5	

^1^Nonparametric Mann-Whitney test: Comparison of two independent populations

^2^Nonparametric Kruskal-Wallis test: Comparison of multiple independent populations

- Gender (*p* = 0.001): higher in females.
- Age group (*p* = 0.001): decreased with increasing age.
- Employment status (*p* = 0.003): highest among trainee nurses.
- Work shift (*p* = 0.001): highest in rotating shifts.
- General health (*p* = 0.013): highest among nurses with poor health.

### Comparison of sleep quality scores

According to Table 5, none of the demographic variables showed significant differences in sleep quality (all *p* > 0.05). This indicates that poor sleep quality is widespread across all subgroups.

**Table 5:** Comparison of sleep quality scores across demographic variables

Variable	Category	Mean	SD	Mean Rank	Sig.
Gender	Female	8.7	2.9	115.42	0.066¹
	Male	7.8	2.9	98.70	
Age group	< 30 years	8.7	3.2	115.03	0.618²
	30–39 years	8.2	2.8	106.44	
	≥ 40 years	8.7	3.1	114.70	
Marital status	Single (widowed/divorced included)	8.2	3.1	105.20	0.397¹
	Married	8.6	2.9	112.70	
Education level	Bachelor’s	8.4	3.1	108.50	0.470¹
	Master’s	8.5	2.4	116.40	
Employment status	Permanent	8.5	2.9	111.90	0.739²
	Contractual	7.9	2.8	100.80	
	Temporary	8.6	2.6	114.80	
	Trainee	8.7	3.6	109.30	
Work shift	Rotating	8.5	2.9	110.30	0.914²
	Morning	8.2	4.2	102.90	
	Evening	8.4	2.1	112.70	
General health status	Poor	8.9	2.1	124.60	0.103²
	Moderate	8.7	3.2	114.10	
	Good	8.3	2.7	108.80	
	Very good	6.6	2.2	72.50	

^1^Nonparametric Mann-Whitney test: Comparison of two independent populations

^2^Nonparametric Kruskal-Wallis test: Comparison of multiple independent populations

### Comparison of occupational burnout scores

As shown in Table 6:

**Table 6:** Comparison of occupational burnout scores across demographic variables

Variable	Category	Mean	SD	Mean Rank	Sig.
Gender	Female	48.2	16.6	104.05	0.045¹
	Male	51.9	15.1	122.40	
Age group	< 30 years	46.8	15.8	—	0.074²
	30–39 years	49.2	16.9	—	
	≥ 40 years	54.8	12.5	—	
Marital status	Single (incl. widowed/divorced)	48.5	17.9	105.20	0.396¹
	Married	49.9	15.2	112.70	
Education level	Bachelor’s	49.2	15.9	110.30	0.878¹
	Master’s	50.2	17.2	108.60	
Employment status	Permanent	46.5	16.5	—	0.018²
	Contractual	53.5	11.9	—	
	Temporary	50.5	18.5	—	
	Trainee	56.3	14.1	—	
Work shift	Rotating	49.6	17.0	—	0.795²
	Morning	46.6	6.1	—	
	Evening	48.3	7.0	—	
General health status	Poor	59.2	16.4	—	0.001²
	Moderate	51.9	15.9	—	
	Good	46.6	14.9	—	
	Very good	38.0	17.9	—	

^1^One-way ANOVA test: Comparing the means of several independent populations

^2^Nonparametric Mann-Whitney test: Comparing two independent populations

- Gender: slightly higher burnout in men (*p* = 0.045).
- Employment status: highest burnout among contractual and temporary staff (*p* = 0.018).
- General health: burnout decreased as health improved (*p* = 0.001).

### Correlations among alarm fatigue, sleep quality, and burnout

Spearman correlation analysis revealed weak but statistically significant positive associations among the study variables. Alarm fatigue was weakly correlated with poor sleep quality (*r* = 0.230, *p* = 0.001) and occupational burnout (*r* = 0.166, *p* = 0.014). Poor sleep quality also showed a weak positive correlation with occupational burnout (*r* = 0.175, *p* = 0.010). These findings indicate that although higher alarm fatigue was associated with poorer sleep quality and greater occupational burnout, the magnitude of these relationships was small and should be interpreted cautiously.

The correlation coefficients ranged from 0.166 to 0.230, indicating small effect sizes. Therefore, although the associations were statistically significant, their practical and clinical significance should be interpreted cautiously.

### Multivariable linear regression analysis

Multivariable linear regression analyses were conducted to examine whether the associations among alarm fatigue, sleep quality, and occupational burnout remained significant after adjustment for demographic and occupational covariates. In the model with alarm fatigue as the dependent variable, sleep quality (B = 0.342, β = 0.166, *p* = 0.008) and occupational burnout (B = 0.081, β = 0.212, *p* = 0.002) remained independently associated with alarm fatigue after adjustment for age, gender, employment status, work shift, monthly number of shifts, and self-rated general health status. The model explained 29.8% of the variance in alarm fatigue (R²=0.298, adjusted R²=0.260).

In the model with sleep quality as the dependent variable, alarm fatigue (B = 0.099, β = 0.202, *p* = 0.008) and occupational burnout (B = 0.039, β = 0.212, *p* = 0.005) remained significant independent predictors. Monthly number of shifts was also positively associated with poorer sleep quality (B = 0.056, β = 0.155, *p* = 0.030). This model explained 14.6% of the variance in sleep quality (R²=0.146, adjusted R²=0.100).

In the model with occupational burnout as the dependent variable, alarm fatigue (B = 0.578, β = 0.220, *p* = 0.002) and sleep quality (B = 0.974, β = 0.181, *p* = 0.005) remained significantly associated with occupational burnout after adjustment for covariates. Older age, male gender, contractual, temporary, and trainee employment status were associated with higher burnout scores, whereas better self-rated general health was associated with lower burnout. This model explained 27.2% of the variance in occupational burnout (R²=0.272, adjusted R²=0.233).

Across the three regression models, the adjusted associations among alarm fatigue, sleep quality, and occupational burnout remained statistically significant. However, the standardized coefficients were small to modest, indicating that these relationships should be interpreted cautiously (Table 7).

**Table 7:** Multivariable linear regression models for alarm fatigue, sleep quality, and occupational burnout

Dependent variable	Independent variable	B	SE	β	95% CI for B	*p*-value
Alarm fatigue	Sleep quality	0.342	0.127	0.166	0.090 to 0.593	0.008
	Occupational burnout	0.081	0.025	0.212	0.031 to 0.131	0.002
	Age	-0.331	0.082	-0.281	-0.493 to -0.169	< 0.001
	Male gender	-2.395	0.822	-0.180	-4.015 to -0.775	0.004
	Evening shift	-4.454	1.635	-0.169	-7.678 to -1.231	0.007
	General health status	-1.123	0.552	-0.128	-2.212 to -0.035	0.043
Sleep quality	Alarm fatigue	0.099	0.037	0.202	0.026 to 0.171	0.008
	Occupational burnout	0.039	0.014	0.212	0.012 to 0.066	0.005
	Monthly number of shifts	0.056	0.026	0.155	0.006 to 0.107	0.030
Occupational burnout	Alarm fatigue	0.578	0.182	0.220	0.219 to 0.937	0.002
	Sleep quality	0.974	0.340	0.181	0.304 to 1.645	0.005
	Age	0.736	0.223	0.238	0.296 to 1.175	0.001
	Male gender	5.288	2.214	0.151	0.924 to 9.652	0.018
	Contractual employment	8.431	2.702	0.205	3.104 to 13.758	0.002
	Temporary employment	6.998	2.869	0.164	1.343 to 12.653	0.016
	Trainee employment	13.042	4.050	0.219	5.058 to 21.026	0.002
	Monthly number of shifts	-0.380	0.126	-0.194	-0.629 to -0.131	0.003
	General health status	-3.543	1.471	-0.154	-6.443 to -0.642	0.017

Reference categories were female gender, permanent employment, and rotating shift. Only statistically significant predictors are shown in the table. All models were adjusted for age, gender, employment status, work shift, monthly number of shifts, and self-rated general health status. B = unstandardized regression coefficient; β = standardized coefficient; CI = confidence interval

## Discussion

The present study aimed to investigate the interrelationships among alarm fatigue, occupational burnout, and sleep quality among ICU nurses. The findings indicate that although the majority of nurses experienced low levels of alarm fatigue, a considerable proportion reported poor sleep quality and varying degrees of occupational burnout. Moreover, weak but statistically significant positive correlations were observed among alarm fatigue, sleep quality, and occupational burnout. These findings suggest that the variables are related; however, the small magnitude of the correlations indicates that these associations should be interpreted with caution and should not be considered evidence of strong or causal relationships.

The weak magnitude of the observed correlations is an important consideration. Although the associations among alarm fatigue, sleep quality, and occupational burnout were statistically significant, the correlation coefficients were small, ranging from 0.166 to 0.230. Therefore, these findings should not be interpreted as evidence of strong practical or clinical relationships at the individual level. Rather, they suggest that these variables are modestly related and that other unmeasured occupational, organizational, psychological, and personal factors may explain a substantial proportion of variation in sleep quality and burnout among ICU nurses.

In this study, more than half of the participants reported no alarm fatigue, while approximately one-third experienced mild alarm fatigue and a small proportion reported moderate levels. These findings are somewhat consistent with previous research that reported relatively low to moderate levels of alarm fatigue among ICU nurses [12]. However, other studies have documented higher levels of alarm fatigue in critical care settings [13, 14]. Differences in alarm management systems, workload, staffing ratios, and organizational policies across healthcare institutions may explain these variations. The presence of large numbers of non-actionable alarms in intensive care units has been widely recognized as an important factor associated with alarm fatigue [4, 5]. Continuous exposure to such alarms may be related to reduced sensitivity to alarm signals and potential patient safety concerns; however, the present cross-sectional study cannot determine causal effects.

The results also revealed that alarm fatigue was significantly higher among female nurses compared with male nurses. Although limited studies have examined gender differences in alarm fatigue, it is possible that differences in workload distribution, emotional involvement in patient care, or stress perception may contribute to this finding. In addition, alarm fatigue decreased with increasing age, suggesting that more experienced nurses may develop better coping mechanisms, improved clinical judgment, and greater familiarity with monitoring systems. Similar findings have been reported in previous studies indicating that clinical experience can mitigate the perceived burden of alarms [1, 17].

Another notable finding of the present study was the significant relationship between alarm fatigue and employment status, with nurses holding temporary or trainee positions reporting higher levels of alarm fatigue compared with permanently employed nurses. In addition, nurses working rotating shifts demonstrated significantly higher alarm fatigue than those working fixed shifts. This finding is partially consistent with the previous studies, which reported that nurses working rotational shifts experienced significantly higher alarm fatigue compared with nurses working fixed shifts in ICU, CCU, and emergency departments. However, their study did not identify employment status as a significant predictor of alarm fatigue, which may be due to differences in sample characteristics, organizational structures, or employment policies across healthcare settings [44].

Alarm fatigue was also significantly associated with nurses’ self-reported general health status in the present study, with nurses reporting poorer health experiencing higher levels of alarm fatigue. This finding suggests that nurses’ physical and psychological well-being may influence how they perceive and cope with environmental stressors such as frequent clinical alarms. In contrast, a study conducted among acute care nurses reported that alarm fatigue was not significantly associated with demographic characteristics, including sex, education level, marital status, or workplace [45]. The discrepancy between findings may be related to differences in study settings, sample sizes, or the variables examined. Nevertheless, both studies highlight that alarm fatigue remains a notable occupational challenge in acute and critical care environments and may interact with various individual and organizational factors.

Regarding sleep quality, the results showed that a large majority of ICU nurses experienced poor sleep quality. This finding aligns with numerous studies demonstrating that nurses, particularly those working in intensive care units and rotating shifts, are highly vulnerable to sleep disturbances [24, 25, 32]. The demanding nature of ICU work, frequent night shifts, high responsibility for critically ill patients, and exposure to stressful clinical environments are all factors that may contribute to impaired sleep quality. Sleep deprivation can negatively affect cognitive performance, decision-making ability, and concentration, thereby increasing the risk of clinical errors [24].

The coexistence of relatively low alarm fatigue with a high prevalence of poor sleep quality is noteworthy. This finding suggests that sleep disturbance among ICU nurses may be influenced by a broader set of occupational and contextual factors beyond alarm fatigue alone. Although alarm fatigue was significantly associated with poor sleep quality, the correlation was weak, indicating that alarm exposure may represent only one component of a wider pattern of sleep disruption. Rotating shifts, night duties, irregular sleep–wake schedules, workload intensity, emotional demands of critical care, and recovery time between shifts may also contribute to poor sleep quality. Therefore, interventions aimed at improving sleep among ICU nurses should not focus exclusively on alarm management, but should also address shift scheduling, workload, rest opportunities, and broader occupational stressors.

Interestingly, no significant differences in sleep quality were observed across demographic or occupational variables such as gender, age, marital status, education level, employment status, or shift type. This may suggest that poor sleep quality is a widespread issue among ICU nurses regardless of their personal characteristics. Similar findings have been reported in previous studies indicating that the stressful nature of intensive care work itself plays a dominant role in sleep disturbances [30].

With respect to occupational burnout, the results showed varying levels across its three dimensions. While emotional exhaustion was generally low to moderate among most participants, depersonalization was moderate to high in a considerable proportion of nurses, and reduced personal accomplishment was reported by the vast majority of participants. These findings suggest that although ICU nurses may maintain emotional resilience to some extent, prolonged exposure to demanding work environments may gradually affect their sense of professional efficacy.

The very high prevalence of reduced personal accomplishment observed in this study warrants careful interpretation. This finding may reflect contextual factors within ICU work environments, including high workload, limited organizational recognition, job insecurity among nonpermanent staff, emotional strain, and limited opportunities for professional growth or feedback. Cultural and organizational expectations may also influence how nurses evaluate their sense of professional efficacy and achievement. In addition, measurement-related factors should be considered. The personal accomplishment dimension of the MBI is conceptually distinct from emotional exhaustion and depersonalization, and lower scores indicate greater burnout in this dimension. Therefore, the high prevalence of reduced personal accomplishment may partly reflect the scoring thresholds used, the cultural adaptation of the instrument, or response patterns in the present sample. Future studies should further examine this dimension using culturally validated cut-off points and, where possible, complementary qualitative approaches.

Although the present study found significantly higher overall burnout levels among male nurses, previous research has reported mixed findings regarding gender differences in burnout. For example, a study conducted among nurse leaders in the United States during the COVID‑19 pandemic showed that female nurse leaders had higher levels of personal burnout, while male nurse leaders reported higher client‑related burnout [46]. These findings suggest that gender differences in burnout may vary depending on the dimension of burnout assessed and the professional roles of nurses. Such variations may be related to differences in role expectations, coping mechanisms, and work responsibilities between men and women in healthcare settings. In addition, the higher burnout observed among nurses with temporary or trainee employment status in the present study highlights the potential impact of job insecurity and limited professional stability on psychological well‑being, which may further interact with gender‑related experiences in the workplace.

Another important finding of the present study was the significant association between general health status and occupational burnout. Nurses who reported poorer perceived health experienced significantly higher levels of burnout compared with those who rated their health more positively. This finding highlights the close interrelationship between nurses’ physical and psychological well-being and their occupational experiences. Working in intensive care units exposes nurses to demanding workloads, high responsibility for critically ill patients, emotional strain, and continuous exposure to stressful stimuli, all of which can negatively affect their overall health status. Previous research has also demonstrated that burnout is strongly linked with both physical and psychological health problems, including chronic fatigue, emotional exhaustion, anxiety, and decreased work capacity [20, 21]. As burnout intensifies, it may further deteriorate nurses’ general health and reduce their ability to cope with workplace stressors, thereby creating a negative cycle that not only affects nurses’ well-being but may also compromise the quality and safety of patient care.

Another contribution of this study is the simultaneous assessment of the associations among alarm fatigue, sleep quality, and occupational burnout among ICU nurses. The findings revealed weak but statistically significant positive correlations among these three variables, indicating that they are related aspects of nurses’ occupational well-being, although the magnitude of these associations was small. Specifically, higher levels of alarm fatigue were associated with poorer sleep quality and greater occupational burnout, while poor sleep quality was also significantly related to increased burnout levels. Interpreted within the JD-R framework, these findings suggest that alarm fatigue may represent a work-related demand associated with poorer sleep quality and greater occupational burnout. Nevertheless, because the observed correlations were weak and the study design was cross-sectional, these results should be interpreted as associations rather than evidence of directional or causal pathways.

Although previous studies have rarely examined alarm fatigue, sleep quality, and occupational burnout simultaneously, existing evidence provides indirect support for the relationships observed in the present study. For example, a study conducted among acute care nurses reported that higher levels of alarm fatigue were accompanied by moderate levels of perceived stress, suggesting that frequent exposure to clinical alarms may increase psychological strain among nurses [45]. Similarly, previous studies found that alarm fatigue among ICU, CCU, and emergency nurses was associated with psychological reactions such as anxiety, irritability, and stress [44]. These findings reinforce the idea that alarm fatigue represents an important occupational stressor in critical care environments. Such psychological strain may be one possible explanation for the observed associations among alarm fatigue, sleep quality, and burnout. However, because stress, anxiety, workload intensity, and staffing levels were not directly measured in the present study, this explanation should be considered speculative. Therefore, although earlier studies have not simultaneously evaluated these three variables, their findings are generally consistent with the results of the present study and collectively highlight the broader psychological and occupational consequences of alarm fatigue in high-intensity clinical settings [44].

The multivariable findings further supported the bivariate results by demonstrating that alarm fatigue, sleep quality, and occupational burnout remained significantly associated even after controlling for key demographic and occupational covariates. Specifically, higher levels of alarm fatigue were independently associated with poorer sleep quality and increased occupational burnout. Likewise, poorer sleep quality was significantly related to higher levels of burnout, indicating a potentially interrelated pattern among these variables. These findings are consistent with previous research indicating that alarm burden, sleep disturbance, and burnout are related occupational health concerns among nurses and critical care staff. Nevertheless, the present study did not directly measure possible mediating variables such as perceived stress, anxiety, workload intensity, nurse-to-patient ratio, staffing adequacy, or objective alarm exposure. Therefore, proposed explanations involving cognitive overload, stress, fatigue, or impaired recovery should be interpreted as plausible hypotheses rather than confirmed mechanisms.

Importantly, the persistence of these associations after adjustment indicates that the relationships observed were not solely attributable to confounding factors such as age, gender, employment status, work shift, monthly workload, or perceived general health. However, the magnitude of both the bivariate correlations and standardized regression coefficients was small to modest. Therefore, the findings should not be interpreted as indicating strong clinical effects at the individual level. Rather, they suggest that alarm fatigue, sleep quality, and burnout are statistically related dimensions of occupational strain, while other unmeasured organizational, psychological, and workload-related factors may play a substantial role. Moreover, due to the cross-sectional design of the study, it is not possible to determine the directionality or causal nature of these associations. Therefore, the findings should be interpreted as adjusted correlations rather than evidence of causal pathways, and longitudinal studies are recommended to further clarify these relationships.

These findings are consistent with previous research indicating that excessive alarm exposure, psychological stress, mental fatigue, poor sleep quality, and burnout are related occupational health concerns among nurses [1, 13]. However, the present study did not assess temporal ordering, indirect effects, or mediation pathways. Therefore, alarm fatigue, sleep quality, and burnout should be interpreted as co-occurring and related aspects of occupational strain rather than as variables within a confirmed causal pathway.

From a clinical and human-factors perspective, these findings support considering alarm fatigue as part of a broader sociotechnical work system in intensive care units. Recent human-factors evidence indicates that nurses’ and physicians’ interactions with monitoring systems are continuous, workflow-dependent, and often shaped by local alarm-management practices. Therefore, alarm customization should not be framed only as an individual nurse responsibility. Rather, it requires unit-level policies, interprofessional collaboration, technical support, staff training, patient-specific adjustment of thresholds, and routine review of alarm data. This interpretation is consistent with international alarm-management guidance and recent evidence on monitor customization. However, because the present study did not evaluate an alarm-management intervention or objectively measure alarm exposure, these implications should be interpreted as practice considerations informed by the current findings and previous literature, not as direct evidence of intervention effectiveness [7–11, 47].

Overall, the results suggest that alarm fatigue should be considered not only as a technological issue but also as a human-factors and occupational health concern. Although the present study cannot determine causal effects, the observed associations indicate that alarm fatigue, sleep quality, and burnout may be relevant considerations when designing supportive and safe clinical environments in intensive care units.

Although multivariable regression analyses were performed to adjust for selected demographic and occupational covariates, residual confounding remains possible. Factors such as nurse-to-patient ratio, workload intensity, unit-level alarm burden, psychological distress, medication use, and institutional alarm management policies were not fully assessed. In addition, because the study was cross-sectional, the adjusted regression models cannot determine the temporal or causal direction of the relationships among alarm fatigue, sleep quality, and occupational burnout.

## Conclusions

The present study demonstrated that ICU nurses experience varying levels of alarm fatigue and occupational burnout, while a substantial proportion report poor sleep quality. The findings revealed weak but statistically significant positive relationships among alarm fatigue, sleep quality, and occupational burnout, indicating that these variables are associated, although the strength of these associations was small. Specifically, higher levels of alarm fatigue were associated with poorer sleep quality and greater occupational burnout, while sleep disturbances were also related to increased burnout. These results suggest that workplace stressors and nurses’ psychological and physical well-being are related in intensive care settings; however, the strength of these associations was small and causal pathways cannot be inferred.

Alarm fatigue was significantly associated with several demographic and occupational characteristics, including gender, age, employment status, shift work, and perceived general health status. Similarly, occupational burnout showed significant associations with gender, employment status, and general health status. In contrast, sleep quality did not differ significantly across most demographic variables, suggesting that sleep disturbance may be a widespread issue among ICU nurses regardless of personal characteristics. Taken together, these findings emphasize that both individual and organizational factors contribute to nurses’ exposure to alarm fatigue and burnout.

Despite providing valuable insights, this study has several limitations that should be considered. First, the cross-sectional design restricts the ability to establish causal relationships among alarm fatigue, sleep quality, and occupational burnout. In addition, although the observed correlations were statistically significant, their effect sizes were small. Therefore, the clinical and practical significance of these associations should be interpreted cautiously. Therefore, longitudinal or prospective studies are required to clarify the directionality and causal mechanisms of these relationships. Second, data were collected using self-reported questionnaires, which may introduce response bias or subjective interpretation and may not fully reflect objective measures of sleep quality, burnout, or exposure to clinical alarms. In addition, because all main variables were measured using self-report instruments at a single time point, the possibility of common-method bias should be considered. Third, the study was conducted in selected hospitals within a specific geographical region of Iran, which may limit the generalizability of the findings to other healthcare settings or countries with different healthcare systems and organizational structures. Fourth, although census sampling was attempted, 27.0% of eligible nurses were not included in the final analysis because they declined participation or returned incomplete questionnaires. Since information on nonrespondents was not available, the possibility of nonresponse bias should be considered when interpreting the findings. Additionally, although multivariable regression analyses adjusted for selected demographic and occupational covariates, residual confounding remains an important methodological limitation. Several potentially influential factors, including workload intensity, nurse-to-patient ratios, ICU type, staffing adequacy, psychological distress, perceived stress, anxiety, recovery time between shifts, objective alarm exposure, and organizational alarm management policies, were not comprehensively measured or adjusted for. Therefore, the observed adjusted associations may still be influenced by unmeasured confounding factors. Similarly, although the discussion proposes possible mechanisms linking alarm fatigue, sleep quality, and occupational burnout, these mechanisms were not directly tested and should be interpreted as hypotheses for future research rather than confirmed pathways.

Based on these findings, several practical implications can be considered. The present study demonstrates adjusted associations among alarm fatigue, sleep quality, and occupational burnout, but it does not provide direct evidence that specific organizational interventions will improve sleep quality or reduce burnout. Therefore, the following recommendations should be interpreted as broader practice implications informed by the current findings together with existing literature on alarm management, shift work, and nurse well-being. Healthcare organizations may consider reviewing alarm management practices, reducing non-actionable alarms, and providing education on appropriate monitoring device use. In addition, strategies related to staffing adequacy, shift scheduling, rest opportunities, and workplace wellness programs may be relevant for supporting ICU nurses’ well-being. However, the effectiveness of these interventions should be evaluated in future longitudinal or interventional studies.

Future research should adopt longitudinal or experimental designs to better explore causal relationships among alarm fatigue, sleep quality, and occupational burnout. Studies involving larger and more diverse samples from different regions and healthcare systems would enhance the generalizability of findings. Moreover, incorporating objective measurements—such as actigraphy for sleep assessment or physiological indicators of stress—could provide more comprehensive and accurate evaluations of these variables.

Overall, the findings suggest that alarm fatigue, poor sleep quality, and occupational burnout are related occupational health concerns among ICU nurses. While alarm management and workforce-related strategies may be relevant areas for organizational attention, future interventional studies are needed to determine whether such strategies can improve sleep quality, reduce burnout, or enhance patient safety outcomes.

## Data Availability

All data generated or analysed during this study are included in this published article.
